# Follicular regulatory T cells eliminate HIV-1-infected follicular helper T cells in an IL-2 concentration dependent manner

**DOI:** 10.3389/fimmu.2022.878273

**Published:** 2022-11-07

**Authors:** Matthew T. Ollerton, Joy M. Folkvord, Andriana La Mantia, David A. Parry, Amie L. Meditz, Martin D. McCarter, Richard T. D’Aquila, Elizabeth Connick

**Affiliations:** ^1^ Department of Medicine, Division of Infectious Diseases, University of Arizona, Tucson, AZ, United States; ^2^ College of Medicine, University of Arizona, Tucson, AZ, United States; ^3^ Department of Otolaryngology, University of Arizona, Tucson, AZ, United States; ^4^ Department of Medicine, Division of Infectious Diseases, University of Colorado, Aurora, CO, United States; ^5^ Department of Surgery, Anschutz Medical Campus, University of Colorado Denver, Aurora, CO, United States; ^6^ Feinberg School of Medicine, Northwestern University, Chicago, IL, United States

**Keywords:** HIV1, IL-2, B cell follicle, follicular helper T (Tfh) cell, follicular regulatory T cell

## Abstract

Follicular helper CD4^+^ T cells (TFH) are highly permissive to HIV and major foci of virus expression in both untreated and treated infection. Follicular regulatory CD4^+^ T cells (TFR) limit TFH numbers and function *in vitro* and *in vivo*. We evaluated the hypothesis that TFR suppress HIV replication in TFH using a well-established model of ex vivo HIV infection that employs tonsil cells from HIV uninfected individuals spinoculated with CXCR4- and CCR5-tropic HIV-GFP reporter viruses. Both CXCR4 and CCR5-tropic HIV replication were reduced in TFH cultured with TFR as compared to controls. Blocking antibodies to CD39, CTLA-4, IL-10, and TGF-beta failed to reverse suppression of HIV replication by TFR, and there were no sex differences in TFR suppressive activity. TFR reduced viability of TFH and even more so reduced HIV infected TFH as assessed by total and integrated HIV DNA. Exogenous IL-2 enhanced TFH viability and particularly numbers of GFP+ TFH in a concentration dependent manner. TFR reduced productively infected TFH at low and moderate IL-2 concentrations, and this was associated with decreases in extracellular IL-2. Both IL-2 expressing cells and larger numbers of FoxP3+CD4+ cells were detected in follicles and germinal centers of lymph nodes of people living with HIV. TFR may deplete TFH *in vivo* through restriction of IL-2 and thereby contribute to decay of HIV expressing cells in B cell follicles during HIV infection.

## Introduction

Follicular T helper cells (TFH) are vital to the humoral immune response generated within germinal centers of B cell follicles during infections (1). They facilitate affinity maturation and class switching of antibodies through direct interactions with germinal center B cells (2–4) as well as the production of cytokines including IL-21 and IL-4 (5–8). TFH are major producers of HIV-1 (HIV) and SIV during chronic, untreated infection (9–12), and also serve as important sources of replication competent virus in treated individuals (13–15). Multiple factors likely predispose TFH to HIV and SIV infection and replication including their location in germinal centers adjacent to follicular dendritic cells (FDC) that harbor infectious virus on their surfaces (16–18), heightened permissiveness to HIV (19), and low concentrations of virus-specific CD8^+^ T cells in B cell follicles (11, 12).

Follicular T regulatory cells (TFR) are a population of CD4^+^ T cells found in B cell follicles and germinal centers that directly impair TFH function and proliferation and limit germinal center responses (20–22). TFR express many of the quintessential markers characteristic of TFH including CXCR5, PD-1, and Bcl-6, as well as the regulatory molecules FoxP3, CTLA-4, and high levels of the IL-2 receptor CD25. TFR likely originate from thymic regulatory CD4^+^ T cells (23), although a small population may differentiate from peripheral FoxP3^-^ cells (24). Regulatory T cells repress activation of CD4^+^ T cells through various mechanisms including reduction of extracellular ATP (25), blockade of costimulatory activation (26), release of TGF-β (27) and IL-10 (28), and sequestration of IL-2 (29). Mechanisms by which TFR regulate TFH, however, are not well understood. We have previously shown that TFR expand numerically in untreated HIV and SIV infection *in vivo*, and impair expression of ICOS, IL-4, and IL-21 in TFH in the context of ex vivo HIV infection (30). Whether TFR modulate HIV replication in TFH is unknown.

Given the known suppressive effects of TFR on multiple functions of TFH, we posited that TFR would suppress HIV replication within TFH. We evaluated the impact of TFR on HIV replication in TFH using a well-established tonsil model of HIV infection that has been shown previously to recapitulate multiple aspects of HIV infection in secondary lymphoid tissues (19, 30, 31). This model employs infection of cells with an NL4-3 based X4-tropic or YU2 based R5-tropic HIV green fluorescent protein (GFP) reporter virus for which GFP expression was previously demonstrated to be tightly linked to HIV expression (32). We observed that TFR reduced HIV replication in TFH, but these effects were not mediated by CD39, CTLA-4, IL-10, or TGF-β. TFR reduced TFH viability, particularly HIV-infected TFH viability. IL-2 was shown to promote both TFH cell survival and HIV replication in TFH. TFR suppressed TFH viability and HIV replication at low and moderate concentrations of IL-2, and this was related to reductions in concentrations of extracellular IL-2. Both IL-2 expressing cells and FoxP3+CD4+ cells were detected in follicles and germinal centers of lymph nodes from individuals with untreated HIV infection with median ratios of 1:28 and 1:10, respectively. These findings suggest that TFR mediated sequestration of IL-2 may limit HIV expressing cells by reducing viability of HIV-infected TFH.

## Materials and methods

### Human subjects

Tonsils were obtained from children at low risk for HIV infection who had undergone routine tonsillectomy. Use of tonsil specimens for these studies was reviewed by the University of Arizona Institutional Review Board and determined to not constitute human subjects research, in accordance with guidelines issued by the Office of Human Research Protections and, consequently, informed consent was not required.

Archived inguinal lymph nodes from untreated people living with HIV without AIDS, obtained as previously described (33, 34), were used with permission by the University of Arizona’s IRB.

### HIV reporter virus

293T cells were cultured in DMEM (Gibco) supplemented with 10% FBS and Primocin (*Invivo*gen) and were transfected with X4-tropic pNLENG1-IRES (32) or R5 tropic pNLYUV3-GFP (35) virus plasmid using Lipofectamine 2000 (ThermoFisher). Supernatant was collected 2 days post transfection, centrifuged at 800 xg, and stored in 500 µl aliquots at -80°C prior to use.

### Tonsil TFH and TFR cell isolation and culture

Tonsil CD4^+^ T cells were enriched from disaggregated tonsil tissue using negative selection (STEMCELL Technologies). CD4^+^ T cells were labeled with CD3-AF700 (clone SP34-2, BD), CD8-BV510 (Clone RPA-T8, BioLegend), CXCR5-PE (Clone MU5UBEE, eBioscience), CD25-PECy7 (Clone BC96, Tonbo), and 7-AAD (Tonbo) and sorted using a FACSAriaIII cell sorter equipped with an 85 μm nozzle into TFR (CD3^+^CD8^-^CXCR5^+^CD25^+^7-AAD^-^) and TFH (CD3^+^CD8^-^CXCR5^+^CD25^-^7-AAD^-^) populations. A minimum of 4 x 10^6^ TFH were spinoculated with X4-tropic or R5-tropic GFP reporter virus and labeled with violet proliferation dye 450 (VPD) (Becton Dickinson). Spinoculated VPD^+^TFH were cultured 1:1 with uninfected, unlabeled TFH or TFR at a final concentration of 2x10^6^ cells/ml for five days in Advanced RPMI (Gibco) supplemented with 10% heat inactivated FBS, 1X nonessential amino acids (Gibco), 1X Glutamax (Gibco), 1X Primocin (*In vivo*gen), (Advanced R-10) in the presence of 5 μM saquinavir (Sigma). IL-2 (NIH AIDS reagent program) (36), IL-6 (PeproTech), and 10 μg/ml blocking antibodies to CD39 (Clone A1, Bio-Rad), CTLA-4 (Clone BE0099, BioXcell), IL-10 (Clone JES3-9D7, Biolengend), or TGF-β (Clone 19D8, Biolegend) were added where indicated.

For studies evaluating the impact of media on sex differences, cells were cultured in Advanced R-10 as indicated above with the following alterations: 10% charcoal-stripped FBS (Sigma), 1 μM estrogen receptor antagonist ICI 182,780 (Sigma), or 300 pg/ml β-estradiol (Sigma) where indicated.

### Quantitative polymerase chain reaction for HIV DNA quantification

Live VPD^+^TFH (VPD^+^7-AAD^-^) were sorted after 5-day coculture with unlabeled, uninfected TFH or TFR. DNA was isolated using a Puregene kit (Qiagen). Cellular DNA was quantified by QPCR for cell equivalents of DNA per volume as described previously (37). Total and integrated DNA was quantified as described previously (38) using equal quantities of cellular DNA.

### Flow cytometry

Cells were washed in PBS and resuspended in PBS and Ghost Dye Red 780 (Tonbo). After 2 minutes, antibodies in PBS, and where appropriate, Brilliant Violet Stain Buffer (BD) were added and incubated for 30 minutes in the dark at room temperature. Cells were washed in PBS and resuspended in 2% paraformaldehyde in PBS. A minimum of 2.55 x 10^4^ CountBright absolute counting beads (Molecular Probes) were added and used for cell count determination. For phenotyping, the following antibodies were used: CD3-AF700 (Clone SP34-2, BD), CD8-BV510 (Clone RPA-T8, BioLegend), PD-1-APC or BV785 (Clone EH12.1H7, BioLegend), CXCR5-PE (Clone MU5UBEE, eBioscience), CD25-PECy7 (Clone BC96, Tonbo), CTLA-4-PerCP-eFluor710 (Clone 14D3, ThermoFisher), GITR-PE-eFluor610 (Clone eBioAITR, eBioscience), LAG-3-Superbright 702 (Clone 3DS223H), and CD39-BV711 (Clone TU66, BD). Cells were analyzed on a Fortessa flow cytometer, and data analyzed using FloJo v10.

For intracellular staining, in place of 2% PFA, cells were fixed and permeabilized with FoxP3/Transcription factor staining buffer kit (Tonbo) according to manufacturer’s instructions. Labeling of intracellular FoxP3 and CTLA-4 was performed using the following antibodies: Foxp3-APC (Clone PHC101, eBioscience) and CTLA-4-PerCP-eFluor710 (Clone 14D3, ThermoFisher) for 30 minutes. Cells were washed, suspended in PBS and analyzed on a Fortessa flow cytometer and data analyzed using FloJo v10.

### Intracellular cytokine staining

Cryopreserved tonsil cells were thawed and cultured in Advanced R-10 overnight. 2ml PBS was added and samples were centrifuged at 300xg for 10 minutes. Cell pellets were resuspended in Advanced R-10 supplemented with 50 ng/ml PMA (Sigma), 1 μg/ml ionomycin (Sigma), and monensin (eBioscience) and cultured 5 hours. Samples were washed and stained as indicated for extracellular proteins, then fixed and permeabilized with Cytofix/Cytoperm (BD) according to manufacturer’s instructions. Samples were stained for 30 minutes with either IL-10-AF647 (Clone JES3-9D7, Biolegend) or TGF-β (Clone TW4-2F8, Biolegend), and analyzed on a Fortessa flow cytometer. Gates for IL-10 and TGF- β were determined using unstimulated, monensin treated samples as controls.

### IL-2 ELISA

On day 5 of infection, cell cultures were centrifuged at 800 g for 10 minutes and supernatant was collected and stored at -80°C. IL-2 ELISA (Sigma) was performed according to the manufacturer’s instructions using diluted supernatant. Samples were analyzed on a plate reader at 450 nm and IL-2 concentrations were calculated using IL-2 standards provided.

### *In situ* hybridization and immunofluorescent staining of lymph nodes from people living with HIV

Six micron sections of frozen inguinal lymph nodes were thaw mounted onto glass slides and fixed in 4% paraformaldehyde for 30 min. After blocking with hydrogen peroxide, sections were stained for IL-2 and CD4 RNA using multiplexed fluorescent *in situ* hybridization (ACDbio). Following *in situ* hybridization, sections were incubated overnight with antibodies to FoxP3 (clone 236A/E7, Invitrogen) and either CD20 (Rabbit pAb, Abcam) or Ki67 (Rat clone OT1567, Abcam) on adjacent sections. Appropriate fluorescent secondary antibodies (Invitrogen) were added and all sections were counterstained with DAPI. Whole sections were imaged on an Aperio slide scanning system (Leica) at 40X.

### Quantitative image analysis

Using Aperio Imagescope (v 12.4.0.5043, Leica), total, follicular (based on CD20 staining) and germinal center (based on Ki67 staining) areas were defined. IL-2 RNA positive cells were counted and categorized as extrafollicular, follicular, or germinal center IL-2 positive cells and their frequency determined by quantitative image analysis. Percentages of IL-2 positive cells that co-expressed CD4 were also determined. In follicles or germinal centers where there were IL-2+ cells, FoxP3+CD4+ cells were counted and the ratio of IL-2+ cells to FoxP3+CD4+ cells was determined. A final ratio was obtained by averaging data from each IL-2 containing follicle or germinal center for each lymph node.

## Results

### TFR inhibit HIV replication in TFH

**Figure 1:**
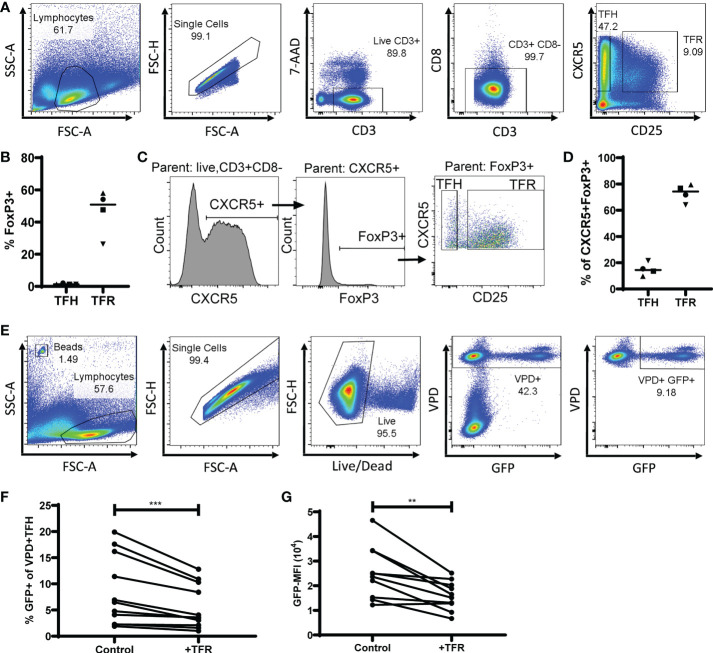
TFR reduce CXCR4-tropic HIV replication in TFH. **(A)** A representative gating strategy for sorting TFH (CD3+CD8-CXCR5+CD25^lo^) and TFR (CD3+CD8-CXCR5+CD25^hi^) from CD4-enriched disaggregated tonsil cells. **(B)** FoxP3 expression in TFH and TFR populations (n=4). **(C)** Representative gating strategy to determine CXCR5^+^FoxP3^+^ expression in TFH and TFR populations. **(D)** Percentages of CXCR5^+^FoxP3^+^ cells gated as TFH or TFR (n=4). **(E)** Representative gating strategy for detection of VPD^+^ TFH and GFP expression in TFH cocultures. TFH were spinoculated with CXCR4-tropic HIV GFP reporter virus, labeled with VPD, and cultured for five days at a ratio of 1:1 with uninfected, unlabeled TFH (control) or TFR in media supplemented with 10 IU/ml IL-2 and 5 μM saquinavir (n=11). After five days, percentages of GFP^+^VPD^+^ TFH **(F)** and GFP-MFI **(G)** of GFP^+^VPD^+^ TFH were determined. Statistical analyses were performed using Wilcoxon matched-pairs tests using Graphpad Prism v8 and significance indicated: **p<0.01; ***p<0.001.

To determine the impact of TFR on HIV replication in TFH, both populations were sorted from tonsils, TFH were spinoculated (39) with the CXCR4-tropic GFP reporter virus (19), labeled with violet proliferation dye (VPD), and cultured for five days with unlabeled TFR or unlabeled, uninfected TFH as a control. Cultures were performed in the presence of saquinavir, to prevent spreading infection, and low concentrations of IL-2 (10 IU/ml). A representative example of flow cytometry gating used for sorting TFH (CD3^+^CD8^-^CXCR5^+^CD25^lo/-^) and TFR (CD3^+^CD8^-^CXCR5^+^CD25^hi^) is shown in **Figure 1A**. Median purity of sorted populations was 98% for TFH (range 91-100%) and 94% for TFR (85-100%). Using this gating strategy, the median ratio of TFR : TFH was 1:7 (range 1:4 to 1:13; n=32). Approximately half of all TFR expressed FoxP3 (median, 51% FoxP3^+^) while very few TFH expressed FoxP3 (median, 1% FoxP3^+^) (**Figure 1B**), as reported previously (19, 30). Furthermore, CXCR5^+^FoxP3^+^ cells were predominantly classified as TFR (median, 74%) compared to TFH (median, 14%) (**Figures 1C, D**) thus validating this sorting strategy. After five days in culture, GFP expression was measured by flow cytometry in VPD^+^TFH as depicted in a representative flow plot (**Figure 1E**). Less than 0.1% of VPD- cells were GFP^+^, demonstrating the efficacy of saquinavir in preventing spreading infection. When compared to controls, TFR cultures reduced percentages of GFP^+^ VPD^+^TFH (median reduction, 36%) (**Figure 1F**) as well as median fluorescence intensity (MFI) of GFP (median reduction, 23%) (**Figure 1G**).

Additional experiments to evaluate the impact of TFR on CCR5-tropic HIV GFP reporter virus expression revealed a similar magnitude of TFR-mediated suppression of HIV replication as seen in CXCR4-tropic HIV GFP reporter virus infection (**Supplementary Figure 1**). Thus, virus tropism did not appear to impact TFR-mediated suppression of HIV replication. Because of the more robust infection seen with CXCR4-tropic virus, subsequent experiments were performed using the CXCR4-tropic GFP reporter virus except when noted.

The defined TFH population consisted of both CXCR5^mid^ and CXCR5^hi^ cells. To determine if the impact of TFR on HIV replication in TFH was different between CXCR5^mid^ and CXCR5^hi^ populations, these subsets were sorted, spinoculated with CXCR4-tropic GFP reporter virus, labeled with VPD, and cultured five days with unlabeled control or TFR cells (**Supplementary Figure 2**). Although percentages of GFP+ cells were elevated in CXCR5^hi^ TFH compared to CXCR5^mid^ TFH (p<0.0001), TFR reduced percentages of GFP+ cells similarly for both subsets (p=0.226). The TFH (CD3^+^CD8^-^CXCR5^+^CD25^lo/-^) population was thus used for all subsequent experiments.

### TFR-related suppression of HIV replication is not mediated by CD39, CTLA-4, IL-10, or TGF-β

**Figure 2:**
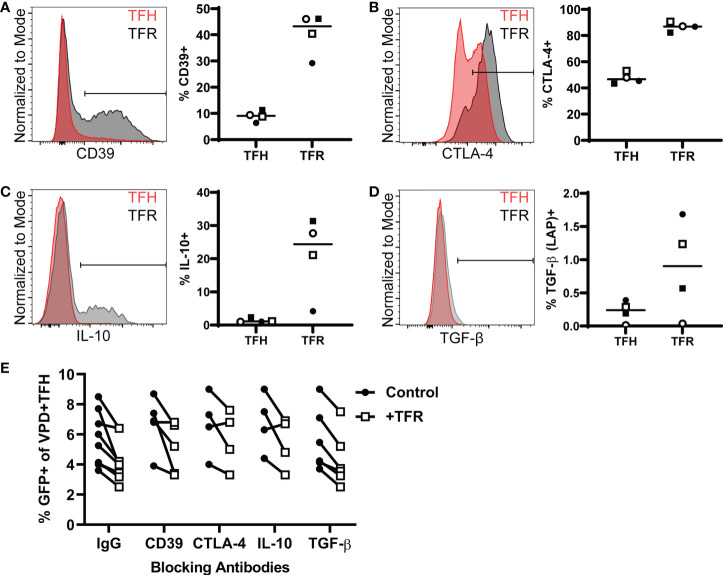
TFR mediated reduction of HIV replication is independent of CD39, CTLA-4, IL-10, or TGF-β. **(A)** CD39 surface expression in TFH and TFR from freshly isolated tonsil CD4^+^ T cells is shown in a representative histogram overlay (left) and TFH and TFR percentages expressing CD39 from four tonsils (right). **(B)** CTLA-4 expression in TFH and TFR from freshly isolated tonsil CD4^+^ T cells is shown in a representative histogram overlay (left) and percentages of TFH and TFR expressing CTLA-4 plotted from four tonsils (right). **(C-D)** Tonsil CD4^+^ T cells were stimulated with PMA/Ionomycin for 5 hours in the presence of Monesin. Cells were fixed, permeabilized, and stained for IL-10 **(C)** or TGF-β **(D)** as shown by a representative histogram (left) and percentages of IL-10^+^ **(C)** or TGF-β^+^ cells **(D)** in TFH and TFR from four tonsils (right). **(E)** TFH were spinoculated with CXCR4-tropic HIV GFP reporter virus, labeled with VPD, and cultured for five days at a ratio of 1:1 with uninfected, unlabeled TFH (control) or TFR in media supplemented with 10 IU/ml IL-2 and 5 μM saquinavir in the presence or absence of blocking antibodies to CD39, CTLA-4, IL-10, or TGF-β. GFP expression was measured after 5 days by flow cytometry according to the gating strategy in **Figure 1**.

TFR exert immunosuppressive effects through the expression of multiple regulatory molecules including CD39 and CTLA-4 (40), and cytokines including IL-10 and TGF-β (41). To determine whether TFR utilize these regulatory mechanisms to inhibit HIV replication in TFH, TFR were first analyzed for expression of these markers. At baseline, a greater percentage of TFR were CD39^+^ (median, 43%) compared to TFH (median, 9%) (**Figure 2A**). Nearly all TFR (median, 87%) and approximately half of TFH (median, 46%) expressed CTLA-4 intracellularly (**Figure 2B**). Some TFR expressed IL-10 (median 24%) upon stimulation with PMA/ionomycin in the presence of monensin, while most TFH did not (median, 1%) (**Figure 2C**). Little TGF-β was expressed by either TFR (median, 0.9%) or TFH (median, 0.2%) (**Figure 2D**). Despite expression of these regulatory molecules by TFR, blocking antibodies to CD39, CTLA-4, IL-10, or TGF- β did not reverse TFR-mediated reductions in percentages of GFP^+^ TFH (**Figure 2E**).

### TFR reduce cell-associated HIV DNA in TFH and TFH viability

**Figure 3:**
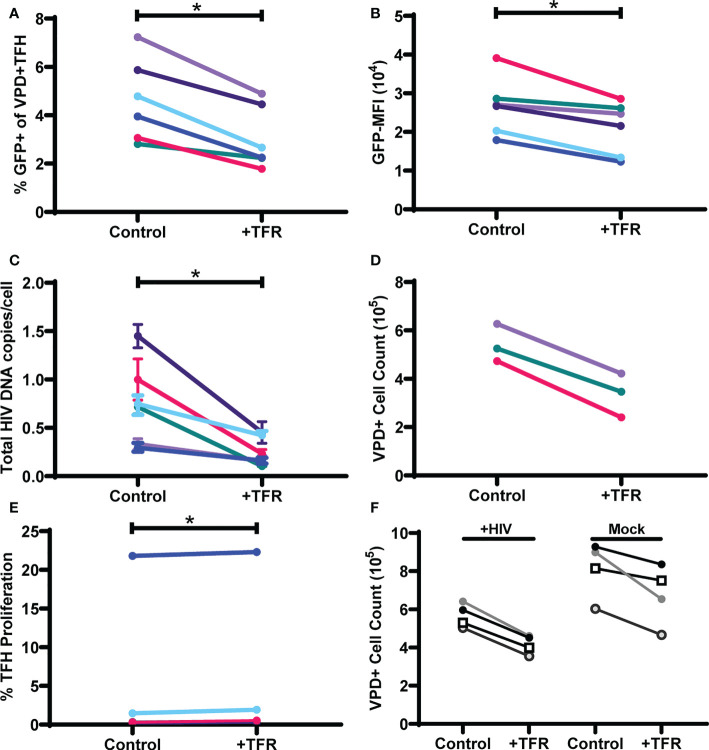
TFR reduce HIV expression in and viability of TFH. TFH were spinoculated with CXCR4-tropic HIV GFP reporter virus, labeled with VPD, and cultured for five days at a ratio of 1:1 with uninfected, unlabeled TFH (control) or TFR in media supplemented with 10 IU/ml IL-2 and 5 μM saquinavir (n=6). Percent GFP^+^ **(A)** and GFP-MFI **(B)** of live VPD^+^ TFH was assessed by flow cytometry after five days using the gating strategy in **Figure 1**. **(C)** DNA was isolated from sorted live VPD^+^ TFH and total HIV DNA was quantified by QPCR. **(D)** In three of the cocultures, absolute cell counting beads were added to determine total VPD^+^ TFH cell counts. **(E)** Percentages of proliferating VPD^+^ TFH were assessed by dye dilution in cocultures after 5 days (n=6). **(F)** In a separate experiment, TFH were spinoculated with CXCR4-tropic as described above. Cell counting beads were added to determine total VPD^+^ TFH cell counts after 5 days in culture (n=4). Statistical analyses were performed using Wilcoxon matched-pairs tests using Graphpad Prism v8 and significance indicated: *p<0.05.

To determine whether TFR-mediated reductions in HIV GFP reporter virus expression were related to reductions in numbers of infected cells rather than inhibition of HIV transcription, total HIV DNA was quantified from six additional tonsils that were spinoculated and cultured for five days in the same way as described above. Live VPD^+^TFH were then isolated on a cell sorter, DNA was isolated, and HIV DNA measured using quantitative PCR (qPCR). As shown previously in **Figure 1**, percentages of GFP^+^VPD^+^TFH (**Figure 3A**) and GFP-MFI (**Figure 3B**) were again consistently reduced in TFR cocultures compared to controls, with median reductions of 36% and 23%, respectively. Total HIV DNA per VPD^+^TFH was markedly decreased in TFR cocultures compared to control cultures with a median reduction of 61% (**Figure 3C**). In two additional experiments, reductions in integrated HIV DNA were observed in the presence of TFR and paralleled reductions seen in total HIV DNA (**Supplementary Figure 3**). Absolute VPD+ cell counts were quantified in three experiments and revealed that viable VPD^+^TFH cells were consistently reduced in TFR cocultures compared to controls with reductions in viable cells ranging from 33-49% (**Figure 3D**). To determine if TFR suppressed TFH proliferation, VPD dye dilution was assessed. With the exception of one subject, few TFH proliferated and TFR did not inhibit TFH proliferation. Indeed, the percentages of cells that proliferated was statistically elevated in wells with TFR compared to controls, although the absolute differences were trivial (median, 0.5% compared to 0.3%) (**Figure 3E**), indicating that TFR-mediated reductions in HIV DNA and TFH numbers could not be accounted for by alterations in TFH proliferation. Collectively, these data indicate that TFR reduce viability of TFH in the context of ex vivo HIV infection and that HIV expressing cells and HIV DNA+ cells are preferentially targeted for depletion.

Because prior ex vivo studies indicated HIV infection activates TFR (30), TFR were assessed for their ability to reduce TFH viability in the presence or absence of HIV infection (**Figure 3F**). Fewer viable TFH were found in HIV-infected cultures compared to uninfected cultures. TFR reduced TFH viability in both HIV infected (median reduction, 26%; range, 24-30%) and uninfected cocultures (median reduction, 16%; range, 8-27%). These results indicated that TFR mediated reductions in TFH viability were not driven by HIV infection.

### Sex does not affect TFR suppression of HIV replication in TFH

Females with HIV infection have lower levels of plasma HIV RNA than males (42–44) and we previously demonstrated that women have lower frequencies of HIV RNA+ cells in lymph nodes as well as produce lower amounts of plasma HIV RNA per infected lymph node cell compared to men (45). To evaluate whether sex differences in viral load could be mediated by sex differences in TFR activity, we sought to test whether there were sex differences in the magnitude of TFR suppression of HIV replication in the tonsil model. Sex differences were not observed in percentages of TFR (median, males 5.9%; females 5.9%; p=0.43) (**Supplementary Figure 4A**). To determine whether media supplemented with conventional bovine serum confounded results, TFH and TFR coculture experiments were performed in the following serum media conditions: conventional FBS, FBS supplemented with an estrogen receptor 1 inhibitor ICI (1μm), charcoal stripped FBS, or charcoal stripped FBS supplemented with β-estradiol (300pg/ml). No sex differences were detected in the TFR mediated reduction of GFP expression when conventional FBS was used (p=0.31). The addition of ICI (male, p>0.99; female, p>0.99), use of charcoal stripped media (male, p=0.13; female, p=0.79), or the addition of β-estradiol (male, p=0.08; female, p>0.99) did not affect the TFR mediated reduction in GFP expression when compared with conventional FBS (**Supplementary Figure 4B**). Next, we combined results from experiments in Figs 1-3 with additional experiments and analyzed them by sex. There were no significant sex differences in percentages of GFP+ TFH in the absence (p=0.89) or presence of TFR (p=0.98) (**Supplementary Figure 4C**), and the magnitude of reduction in percentages of GFP+ cells (median, males 35%, females 26%; p=0.26) did not differ significantly between the two sexes (**Supplementary Figure 4D**). Thus, there was no evidence for sex differences in TFR-mediated elimination of HIV-expressing cells.

### IL-2 enhances HIV replication and viability in TFH

**Figure 4:**
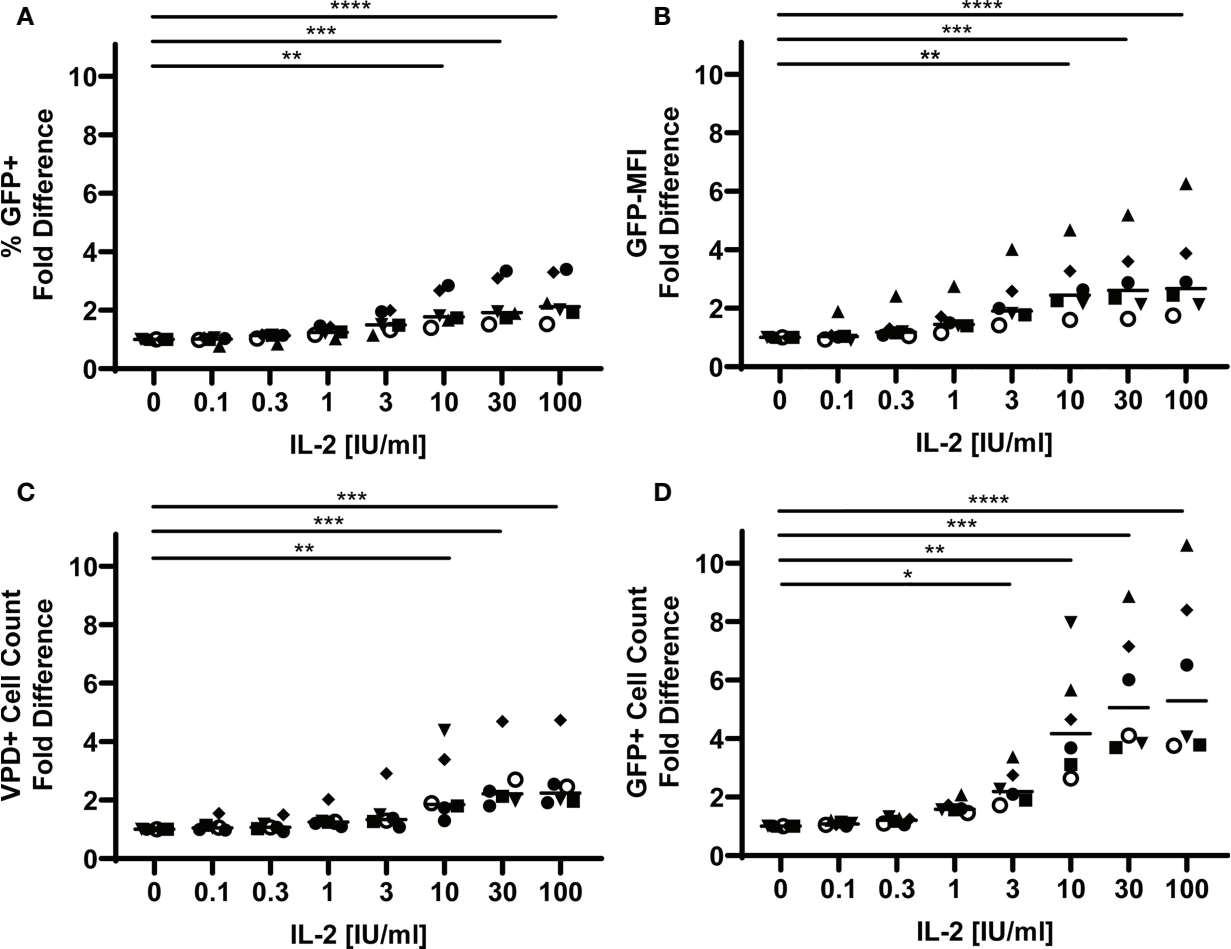
IL-2 promotes HIV replication and TFH viability. TFH were spinoculated with CXCR4-tropic HIV GFP reporter virus, labeled with VPD, and cultured for five days at a ratio of 1:1 with uninfected, unlabeled TFH in media supplemented with 10 IU/ml IL-2 and 5 μM saquinavir (n=6). **(A)** Fold differences of percentages of GFP^+^VPD^+^ TFH were assessed *via* flow cytometry using the gating strategy in **Figure 1**. **(B)** Fold differences of GFP-MFI were determined in GFP^+^VPD^+^TFH. **(C)** Fold differences of VPD^+^TFH and **(D)** GFP^+^VPD^+^TFH cell counts were determined after five days in culture using absolute counting beads. Symbols denote individual tonsils (n=6). Statistical analyses were performed with Friedman’s test and Dunn’s multiple comparison test using Graphpad Prism v8 and significance indicated: *p<0.05; **p<0.01; ***p<0.001; ****p<0.0001.

T regulatory cells have been shown to reduce T cell viability through sequestration of IL-2 (46). To investigate whether TFR also possess this capability, we first determined the impact of IL-2 on GFP expression and TFH viability in the tonsil cell model. VPD-labeled TFH were spinoculated with CXCR4-tropic HIV GFP reporter virus and then cultured with uninfected, unlabeled TFH in the presence of varying concentrations of IL-2. GFP expression and TFH viability were measured after 5 days in culture (**Supplementary Figure 5**) and presented as fold differences for each parameter compared to no IL-2 (**Figure 4**). Approximately two-fold increases in percentages of GFP^+^ TFH and GFP-MFI were observed at IL-2 concentrations equal to or greater than 10 IU/ml (**Figures 4A, B**). Absolute numbers of TFH were also increased at these concentrations by approximately two-fold (**Figure 4C**). GFP+ cell viability, however, was more sensitive to exogenous IL-2 and numbers significantly increased at IL-2 concentrations equal to and greater than 3 IU/ml and were more than five-fold greater in number at the highest IL-2 concentration (**Figure 4D**). Similar patterns were observed in CCR5-tropic HIV GFP reporter virus infected TFH as in CXCR4-tropic GFP reporter virus infected TFH (**Supplementary Figure 6**). Thus, IL-2 enhanced the magnitude of HIV replication in TFH, TFH survival, and particularly GFP^+^ TFH survival in both CXCR4-tropic and CCR5-tropic virus infection.

### IL-6 does not inhibit IL-2-mediated effects on TFH viability and HIV replication, nor TFR mediated inhibition on the same

**Figure 5:**
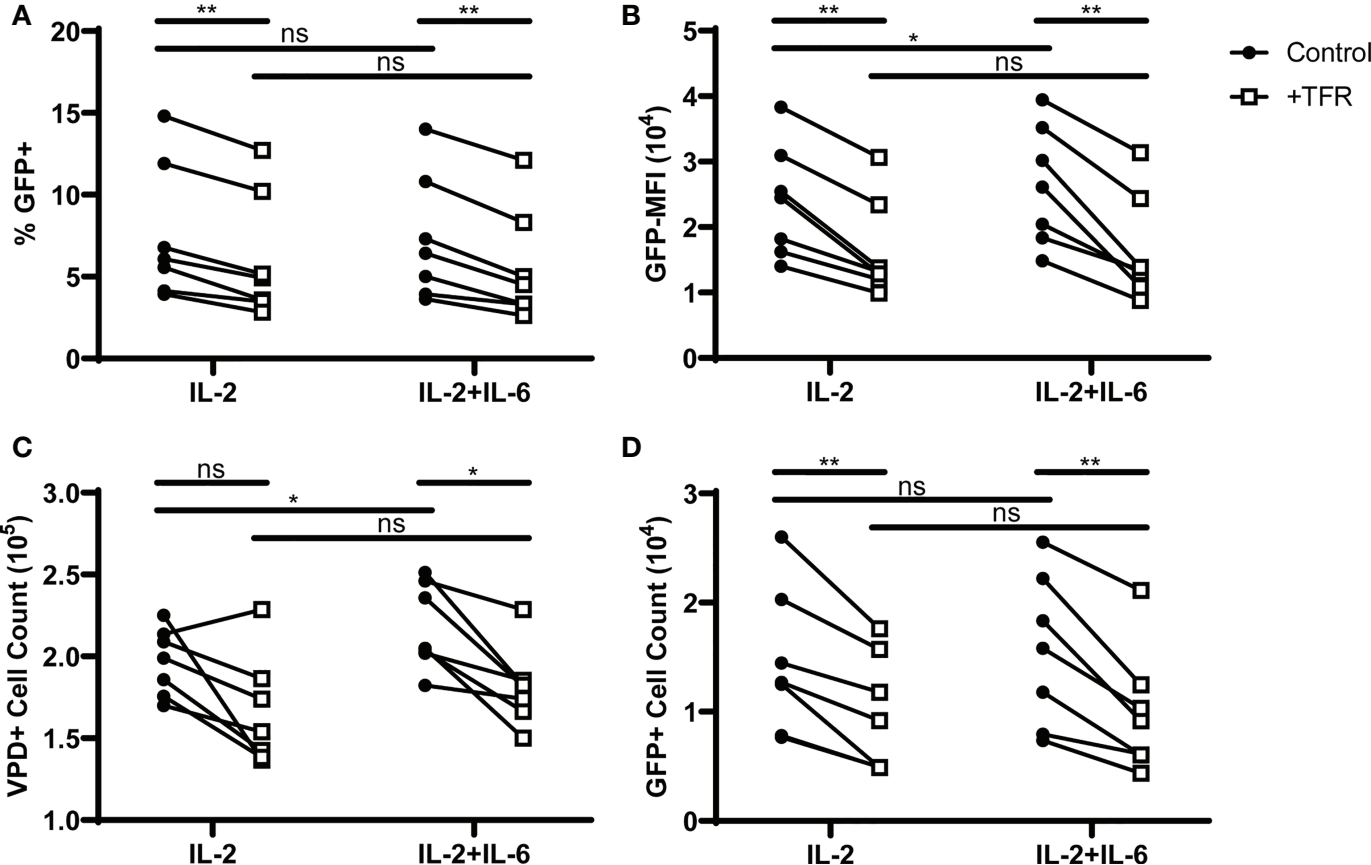
IL-6 does not block IL-2 mediated effects on TFH viability or HIV replication, or TFR-mediated inhibition of TFH viability or HIV replication. TFH were spinoculated with CXCR4-tropic HIV, labeled with VPD, and cultured at a ratio of 1:1 with unlabeled, uninfected TFH (Control) or TFR in the presence of 5 μM saquinavir and 10 IU/ml IL-2 or 10 IU/ml IL-2 and 5 ng/ml IL-6 for 5 days (n=7). **(A)** Percent GFP^+^ expression and **(B)** GFP-MFI in GFP^+^VPD^+^TFH were determined in cocultures with IL-2 or IL-2 and IL-6 using the gating strategy in **Figure 1**. **(C)** Total VPD^+^TFH and **(D)** GFP^+^VPD^+^TFH cell counts were determined in cocultures supplemented with IL-2 or IL-2 and IL-6. All graphs represent data collected from the same six cocultures. Statistical analyses were performed using 2-way ANOVA and Sidak’s multiple comparisons tests (Graphpad Prism v8) and significance indicated: ns not significant; *p<0.05; **p<0.01.

In a mouse model of influenza infection, germinal center TFH were found to be unresponsive to IL-2 through an IL-6 dependent mechanism (47). As the experiments reported here demonstrated that TFH are responsive to IL-2, but were performed in the absence of FDC, which are the major IL-6 producing cells (48), we sought to determine whether IL-6 would enhance HIV replication and TFH viability in IL-2 treated cultures, and negate TFR-mediated suppression of TFH viability and HIV replication. The addition of IL-6 to cultures did not alter the percentage of GFP+ cells (**Figure 5A**) in control wells. However, in control wells treated with IL-2 and IL-6 both GFP MFI (**Figure 5B**) and TFH cell count (**Figure 5C**) were elevated compared to IL-2 alone, suggesting a positive effect of IL-6 on these parameters. In cocultures of TFH and TFR supplemented with 10 IU/ml IL-2 in the presence or absence of 5 ng/ml IL-6, TFR-mediated reductions in percentages of GFP^+^ VPD+TFH were similar in cultures treated with IL-2 alone compared to those with IL-2 and IL-6 (**Figure 5A**). Similarly, TFR-induced reductions in GFP MFI, TFH cell count and GFP+ cell count were unaffected by the presence of IL-6 (**Figures 5B–D**). These results suggest that human TFH maintain responsiveness to IL-2 in the presence of IL-6, and human TFR exert their suppressive functions on TFH viability in the presence of IL-6.

### TFR reductions in HIV-producing TFH are IL-2 concentration dependent

**Figure 6:**
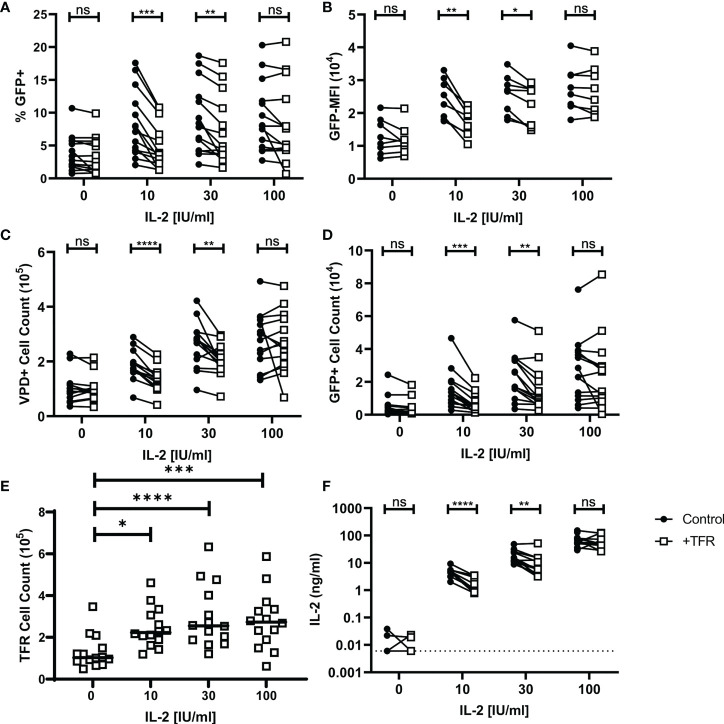
TFR reduction of HIV replication and TFH viability is IL-2 concentration dependent. TFH were spinoculated with CXCR4-tropic HIV, labeled with VPD, and cultured at a 1:1 ratio with unlabeled, uninfected TFH (Control) or TFR for 5 days with 5 μM saquinavir in the absence or presence of 10, 30, or 100 IU/ml IL-2. Percentages of GFP^+^VPD^+^TFH **(A)**, GFP MFI **(B)**, total VPD^+^TFH cell counts **(C)** GFP^+^VPD^+^TFH cell counts **(D)**, and TFR cell counts **(E)** were quantified *via* flow cytometry using absolute counting beads and the gating strategy in **Figure 1** (n=14). **(F)** IL-2 concentrations from 12 of the TFH coculture supernatants were quantified by ELISA. Statistical analyses were performed using 2-way ANOVA and Sidak’s multiple comparisons tests **(A-D, F)**, and Friedman and Dunn’s multiple comparison test **(E)** using Graphpad Prism v8 and significance as indicated: (ns) not significant p>0.05; *p<0.05; **p<0.01; ***p<0.001; ****p<0.0001.

To evaluate whether TFR-mediated reductions in HIV-producing TFH are related to IL-2, TFR and TFH co-culture experiments were repeated in the presence of varying concentrations of IL-2 ranging from 0 to 100 IU/ml IL-2 (**Figure 6**). At concentrations of IL-2 of 10 IU/mL and 30 IU/mL, significant reductions in percentages of GFP+VPD+ TFH, MFI of GFP, and numbers of either VPD+TFH cells or VPD+GFP+ cells occurred in the presence of TFR (**Figures 6A–D**). In contrast, no alterations in these parameters were observed in the presence of TFR at concentrations of IL-2 of 100 IU/mL or when no IL-2 was added. Similar observations were seen in CCR5-tropic infection as in CXCR4-tropic infection (**Supplementary Figure 7**).

TFR cell counts from these experiments were increased in conditions with exogenous IL-2 compared to conditions without IL-2 (**Figure 6E**). However, varying concentrations of IL-2 did not affect TFR cell counts, suggesting no dose effect on TFR. In separate experiments prepared as in **Figure 6**, TFR expression of CD39, GITR, and LAG-3 was measured after five days of culture with varying concentrations of IL-2 (**Supplementary Figure 8**). TFR expression of these effector molecules was lower in cells cultured without IL-2, but again there was no dose effect and no statistically significant differences in expression of these effector molecules among the varying concentrations of IL-2.

IL-2 concentrations were measured in supernatants from control and TFR-treated 5-day cocultures (**Figure 6F**). In the absence of exogenous IL-2, IL-2 concentrations were low in 3 and undetectable in 9 of 12 cocultures. IL-2 supernatant concentrations were significantly reduced in TFR cocultures compared to TFH control cocultures supplemented with 10 IU/ml IL-2 (median, TFR: 2.31 ng/ml; Control: 5.40 ng/ml) and 30 IU/ml IL-2 (median, TFR: 6.14 ng/ml; Control: 13.85 ng/ml). At high concentrations of IL-2, i.e., 100 IU/mL, there was no significant difference between TFR and control co-cultures in terms of IL-2 concentrations. Taken together, these results indicate that TFR reduce TFH viability and HIV replication by reducing extracellular IL-2 concentrations, and that this occurs in the context of low or moderate IL-2 concentrations, but not at high IL-2 concentrations.

### IL-2+ and FoxP3+CD4+ cells are present in lymph node follicles and germinal centers of people living with HIV

**Figure 7:**
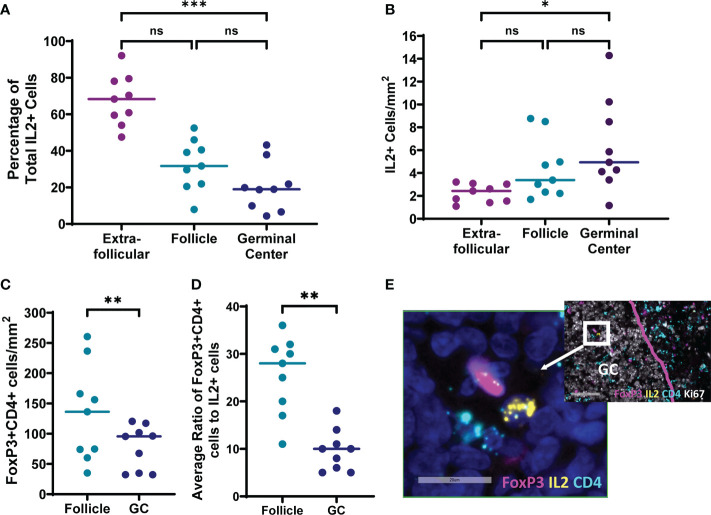
IL-2 expressing cells and FoxP3+CD4+ cells are present in follicles and germinal centers of lymph nodes from individuals living with chronic untreated HIV infection. Six micron sections were stained for IL-2 mRNA, CD4 mRNA, FoxP3, DAPI, and either CD20 or Ki67 (n=9). DAPI was used to determine total tissue area. CD20 and Ki67 staining of adjacent sections was used to locate follicles and germinal centers, respectively. Cells were counted manually and frequencies determined by quantitative image analysis. **(A)** Distribution of IL-2+ cells in extrafollicular, follicular and germinal center regions. Frequencies of **(B)** IL-2+ cells and **(C)** FoxP3+CD4+ cells in each region **(D)** Mean ratios of FoxP3+CD4+ cells to IL-2+ cells were quantified for each follicle or germinal center containing an IL-2+ cell. **(E)** Representative image of a cell expressing IL-2 (yellow) adjacent to a cell expressing FoxP3 (magenta) within a germinal center. Both cells, and multiple other surrounding cells are CD4+ (light blue). DAPI staining of nuclei is shown in dark blue. Statistical analyses were performed using nonparametric Friedman tests **(A, B)** and Wilcoxon tests **(C, D)** using GraphPad Prism v8 and significance as indicated: (ns) not significant p>0.05; *p<0.05; **p<0.01; ***p<0.001.

To evaluate whether IL-2 expressing cells and TFR are found in the follicles and germinal centers of lymph nodes where the majority of TFH reside, inguinal lymph node sections of individuals with untreated chronic HIV infection without AIDS were evaluated for IL-2 and CD4 expression by *in situ* hybridization for mRNA, and FoxP3 by antibody staining (**Figure 7**). All lymph node sections contained IL-2 expressing cells, and the majority (92%; range 79-97%) of IL-2+ cells were CD4+. No IL-2+FoxP3+ cells were detected. The majority (68%; range, 48-92%) of IL-2+ cells were found outside follicles and the fewest IL-2 positive cells were found in the germinal centers (**Figure 7A**). Frequencies of IL-2+ cells/mm^2^ were slightly elevated in germinal centers compared to extrafollicular regions (p=0.0286); however, there was no statistically significant difference in frequencies of IL-2 positive cells between follicles and germinal centers, and follicles and extrafollicular areas (**Figure 7B**). We previously reported that FoxP3+CD4+ cells are most abundant in extrafollicular regions, intermediate in frequency in follicular regions, and least frequent in germinal centers of lymph nodes of people living with HIV (30). We confirmed again that FoxP3+CD4+ cell frequencies were higher in follicles compared to germinal centers (p=0.0039; **Figure 7C**). The median ratio of FoxP3+CD4+ cells to IL-2+ cells was 28:1 (range, 11:1 to 36:1) in follicles and 10:1 (range, 5:1 to 13:1) in germinal centers (**Figure 7D**). FoxP3+CD4+ cells found within follicles and germinal centers were detected adjacent to IL-2+ cells in some instances (**Figure 7E**). Thus, IL-2 expressing cells exist within the follicular and germinal center milieu of lymph nodes in HIV infection with the potential to influence surrounding TFH, and even larger numbers of TFR compared to IL-2 producing cells are present with the capacity to reduce local concentrations of IL-2.

## Discussion

TFH are the primary HIV-producing cells in chronic, untreated infection prior to development of AIDS (9, 10) and also harbor a significant fraction of the latent and expressed HIV reservoir in treated individuals (11, 12). This is the first study to evaluate the impact of TFR on HIV replication in TFH. Using an ex vivo model of HIV infection of tonsil cells with an HIV-GFP reporter virus, we found that GFP expression in TFH, a surrogate marker for HIV replication (32), was reduced in the presence of TFR. Decreases in HIV replication were observed in both CXCR4-tropic and CCR5-tropic HIV infection, were not mediated by the regulatory mechanisms CD39, CTLA-4, IL-10, or TGF-β, and did not differ by sex. IL-2 promoted cell viability and HIV replication in TFH. TFR reduced TFH cell viability and particularly HIV-infected TFH cell viability, and these reductions occurred at low and moderate concentrations of IL-2, i.e., 10 to 30 IU/mL and were directly related to decreased concentrations of IL-2. *In vivo*, both IL-2 expressing cells and TFR were detected in follicles and germinal centers of lymph nodes from people living with HIV, and TFR substantially outnumbered IL-2+ cells. This is the first study to suggest that IL-2 restriction may be a mechanism by which TFR modulate TFH numbers, even in the absence of HIV infection. TFH may be particularly susceptible to IL-2 restriction due to their highly activated state. Furthermore, this is the first study to suggest that IL-2 consumption by TFR could be an important mechanism whereby TFR limit HIV expressing TFH.

A major challenge in the study of TFR is the controversy over their phenotype. TFR were first characterized in mice in 2011 and identified as CD4^+^CXCR5^+^FoxP3^+^ cells (20, 22, 49). Several groups later expanded the TFR phenotype to include CD25 expression (22, 30, 40, 41). Nevertheless, it was subsequently reported that some germinal center TFR do not express CD25, and it has been hypothesized that CD25 expression is lost as TFR become more differentiated and migrate to germinal centers (50, 51). Our data confirmed that nearly all CXCR5^+^FoxP3^+^ cells were CD25^+^, while the vast majority of TFR cells expressed CXCR5 at intermediate levels, corroborating findings by Sayin et al. (40). Most recently, one group reported that tonsils harbor CD4^+^CXCR5^+^CD25^+^ TFR that do not express FoxP3, but produce IL-10 (41). We defined TFR as CD3^+^CD4^+^CXCR5^+^CD25^hi^ cells in our study. On average 50% of these cells expressed FoxP3 and more than 75% of CXCR5^+^FoxP3^+^ expressed CD25^hi^. Thus, the TFR phenotype utilized in this study included the IL10-producing FoxP3^-^ TFR. Although our definition of TFR excluded the putative CD25^lo/-^TFR subset, they constituted on average only 15% of the CXCR5+FoxP3^+^ population in the tonsils we studied.

It is well established that IL-2 is a potent stimulant of peripheral blood CD4+ T cell proliferation and HIV replication in the context of mitogen stimulation. This is the first study, however, to examine the impact of IL-2 on HIV replication in human TFH. Prior studies in mice suggested that high levels of exogenous IL-2 (500 IU/ml) inhibit the differentiation of CD4+ T cells into both TFR and TFH through induction of STAT5 (52, 53), whereas low levels of IL-2 (10 IU/ml) induced a TFH phenotype. Furthermore, in mouse models, TFH produce high levels of IL-2 (54) and are reportedly resistant to autocrine IL-2 stimulation due to IL-6 (47, 54). Our studies indicated that TFH cell survival, and particularly survival of TFH actively replicating HIV, was promoted by exogenous IL-2. Importantly, inclusion of a proliferation marker verified that IL-2 did not cause TFH to proliferate, consistent with the fact that TFH are terminally differentiated effector cells. IL-6 did not compromise the response of TFH to IL-2, and in combination with IL-2 it enhanced TFH cell viability and GFP-MFI. It is unclear whether these differences in the impact of IL-6 on IL-2 responsiveness between human TFH and mouse TFH reflect true differences between the species or are the result of differences in the models used. Our identification of IL-2 expressing cells in the follicles and germinal centers of lymph nodes from people living with HIV further strengthens the biological plausibility that IL-2 may play an important role in survival of TFH and particularly HIV-producing TFH *in vivo*.

Treg consumption of IL-2 as a mechanism to modulate immune responses has been described previously in several mouse models including inhibition of polyclonal activation of CD4+ T cells and induction of apoptosis of effector CD4+ T cells (29, 55), inhibition of development of CD8+ cytotoxic T cell responses (56), and inhibition of maturation of natural killer cells (57). Treg consumption of IL-2 has also been demonstrated to promote induction of TFH in an influenza mouse model (58) as well as TH17 cells (59), both of which mature in the context of low concentrations of IL-2. Importantly, induction and maturation of different T cell phenotypes occurs largely in the extrafollicular regions of lymphoid tissues (60). Tregs outcompete other cells for IL-2 because of high levels of expression of the IL-2 receptor CD25, and IL-2 in turn induces proliferation of Tregs. In a mouse model, IL-2 effects in spleen and lymph node were shown to follow a simple diffusion-consumption model; IL-2 production influenced cells as far as 40-60 μm away, but the distance of IL-2 effects was inversely proportionate to Treg frequency (61). We previously showed that TFH had increased apoptosis in cultures with TFR in the presence of IL-2 (30). We now report that TFR impair survival of TFH in an IL-2 concentration dependent manner. This is also the first study to report that any regulatory T cell inhibits HIV replication through depletion of infected cells. Sequestration of IL-2 by TFR is the most likely mechanism, and was elegantly described in Treg in a mouse model of inflammatory bowel disease (29). Similar to results reported here, the authors observed that culture of CD4^+^ T cells with Tregs resulted in decreased exogenous IL-2 concentrations and increased apoptosis of CD4^+^ T cells. Intriguingly, depletion of Tregs using anti-CD25 antibodies in rhesus macaques with natural virologic control resulted in increased plasma viremia despite augmentation of SIV-specific CD8+ T cell responses (62, 63). These findings are consistent with loss of TFR-mediated depletion of virus-producing cells in B cell follicles, where increased virus-specific CD8+ T cell responses would not be expected to compensate due to their relatively low frequency. Future studies to evaluate the impact of Treg depletion on IL-2 expression *in vivo* and the TFH HIV reservoir in non-human primates could further address this hypothesis.

A limitation of the present study is that the cell culture experiments were performed ex vivo and consequently differed in some respect to *in vivo* conditions. We previously reported that TFR are more permissive to HIV than TFH (31), but in the experiments reported here TFR were protected from infection by addition of saquinavir. Importantly, however, only a minority of TFR are infected *in vivo* (64), and consequently the impact of HIV-infected TFR on the study observations would be expected to be minimal. Our experiments excluded other cells present in the follicular milieu, specifically B cells, follicular dendritic cells, and stromal cells, which could produce cytokines that potentially diminish the impact of IL-2 deprivation. Although we evaluated the impact of exogenous IL-6 on TFR-mediated suppression of HIV replication and found that TFR-mediated suppression of HIV replication persisted, we cannot rule out the possibility that other cytokines in the follicular milieu that were not present in our experiments could diminish the impact of IL-2 deprivation. Finally, although we established that both IL-2 expressing cells and TFR were present in B cell follicles and germinal centers in people living with HIV, the absolute concentrations of IL-2 in these regions are unknown.

Emerging data indicate that despite relative stability of the total HIV DNA reservoir after several years of ART, ongoing decay of the replication competent reservoir occurs and is likely driven by HIV expression (38). It has been assumed that decay of the replication competent reservoir during ART is related to cellular antiviral effectors such as CD8+ T cells and natural killer cells. Nevertheless, neither of these cell types is located in large concentrations within B cell follicles where TFH harbor a large fraction of the expressed HIV reservoir in treated disease (15). Results of our study suggest that TFR may contribute to decay of the HIV reservoir by hindering viability of TFH, particularly those TFH that express HIV. It has been proposed that Treg depletion may be used as a strategy to facilitate HIV cure under the assumption that they operate mechanistically to suppress virus reactivation, and that their depletion results in reactivation of latently infected cells that then can be eliminated by the immune response (65). Indeed, a study in HIV-infected humanized mice revealed that transient depletion of Tregs resulted in transient increases in HIV expression and ultimately modest reductions in the HIV reservoir (66). A significant limitation of most humanized mouse models of HIV infection including the one used in this study, however, is that they fail to recapitulate B cell follicles and germinal centers, where a large fraction of the HIV reservoir resides. Thus, their relevance to *in vivo* infection and particularly the HIV reservoir is questionable. The data presented here suggest that depletion of TFR could promote persistence of HIV producing TFH cells and impede decay of the TFH reservoir during ART. A better understanding of the factors that modulate HIV expression within TFH in the B cell follicle during ART is essential to designing rational HIV cure strategies.

## Data availability statement

The original contributions presented in the study are included in the article/**Supplementary Material**. Further inquiries can be directed to the corresponding author.

## Author contributions

MO, JF, and EC conceptualized and designed the study. MO, JF, and AL collected all data. DP, AM, and MM acquired tissue samples. MO and EC wrote the manuscript. All authors contributed to the article and approved the submitted version.

## Funding

This work was partially funded by the NIH/NIAID grant (P01 AI131346 and Administrative Supplement) and supported by the NCI award (P30 CA023074) to the University of Arizona Cancer Center Flow Cytometry Shared Resource. Support for Andriana La Mantia was provided by T35HL007479 (Witte). Support for Matthew Ollerton was provided by the Moya-Teller Fund. Funding sources had no role in the experimental design, data collection, interpretation of data, or decision to submit the manuscript for publication.

## Acknowledgments

GFP reporter virus plasmid was contributed by Dr. David Levy. Dr. Una O'Doherty provided invaluable guidance regarding DNA quantification assays. Human rIL-2 was provided by Dr. Maurice Gately, Hoffmann - La Roche Inc. through the HIN AIDS Reagent Program, Division of AIDS, NIAID, NIH.

## Conflict of interest

The authors declare that the research was conducted in the absence of any commercial or financial relationships that could be construed as a potential conflict of interest.

## Publisher’s note

All claims expressed in this article are solely those of the authors and do not necessarily represent those of their affiliated organizations, or those of the publisher, the editors and the reviewers. Any product that may be evaluated in this article, or claim that may be made by its manufacturer, is not guaranteed or endorsed by the publisher.

## Supplementary material

Supplementary Figure 1 TFR cause a similar reduction of HIV replication in TFH infected with CXCR4- or CCR5-tropic HIV. TFH were spinoculated with CXCR4- or CCR5-tropic HIV, labeled with VPD, and cultured with uninfected, unlabeled TFH or TFR in media supplemented with 10 IU/ml IL-2 and 5 μM saquinavir. **(A)** Percent GFP^+^ of VPD^+^ TFH and **(B)** GFP MFI of GFP^+^VPD^+^ TFH were determined after 5 days by flow cytometry using the gating strategy in (n=4).

Supplementary Figure 2 TFR reduce CXCR4-tropic HIV replication in CXCR5^mid^ and CXCR5^hi^ TFH. A representative post sort analysis showing CXCR5 and PD-1 expression on **(A)** CXCR5^mid^ and **(B)** CXCR5^hi^ TFH after sorting CD4 enriched disaggregated tonsil cells. **(C)** CXCR5^mid^ and CXCR5^hi^ TFH were spinoculated with CXCR4-tropic HIV, labeled with VPD, and cultured at a 1:1 ratio with unlabeled TFH or TFR in media containing 10 IU/ml IL-2 and 5 μM saquinavir. Percentages of GFP^+^VPD^+^ TFH were determined after five days by flow cytometry using the gating strategy in (n=6). Statistical analysis was performed using 2-way ANOVA using Graphpad Prism v8 and significance indicated: ns not significant.

Supplementary Figure 3 TFR reduce total and integrated HIV DNA in TFH. TFH isolated from tonsils were spinoculated with CXCR4-tropic HIV, labeled with VPD, and cultured at a ratio of 1:1 with uninfected, unlabeled TFH (control), or TFR for 5 days in media supplemented with 10 IU/ml IL-2 and 5 μM saquinavir. DNA was isolated from live sorted TFH. Cellular DNA, total HIV DNA **(A)**, and integrated HIV DNA **(B)** were determined using QPCR (n=2).

Supplementary Figure 4 Sex differences were not observed in percentages of TFR or TFR mediated inhibition of HIV replication in TFH. **(A)** Percentages of TFR from tonsil CD3^+^CD8^-^ cells stained as in were evaluated for sex differences (males, n=16; females, n=14). Statistical analysis was performed using Mann-Whitney test using Graphpad Prism v8 and significance indicated: ns not significant. **(B)** TFH isolated from tonsils of six males and six females were spinoculated with CXCR4-tropic HIV GFP reporter virus, labeled with VPD and cultured at a ratio of 1:1 with uninfected, unlabeled TFH, or TFR for five days in media supplemented with 10 IU/ml IL-2, saquinavir, and either FBS, FBS supplemented with an estrogen receptor inhibitor (ICI), charcoal stripped FBS (csFBS), or csFBS supplemented with β-estradiol. The decrease in percentages of GFP^+^ cells was measured for each condition. **(C)** TFH and TFR were sorted from subjects in (A), spinoculated, labeled, and cultured as indicated in **(B)** with the exception that all cultures were supplemented with FBS, 10 IU/ml IL-2, and saquinavir. Percent GFP^+^ of VPD^+^ TFH was determined after five days using the gating strategy in . Statistical analysis was performed using 2-way ANOVA and Sidak’s multiple comparison test using GraphPad Prism v8 and significance indicated: ns, not significant; ****p<0.0001. **(D)** Sex differences in percent decrease of GFP^+^ in cultures shown in **(C)** were quantified and statistical analysis was performed using Mann-Whitney test using Graphpad Prism v8 and significance indicated: ns not significant.

Supplementary Figure 5 Raw data from showing that IL-2 promotes HIV replication and TFH viability. TFH were spinoculated with CXCR4-tropic HIV, labeled with VPD, and cultured at a ratio of 1:1 with uninfected, unlabeled control TFH cells with varying concentrations of IL-2 and 5 μM saquinavir. **(A)** Percentages of GFP^+^VPD^+^ TFH, **(B)** GFP MFI, **(C)** VPD^+^TFH and **(D)** GFP^+^VPD^+^TFH cell counts were determined by flow cytometry after five days in culture using the gating strategy in . Symbols denote individual tonsils (n=6). Statistical analyses were performed with Friedman’s test and Dunn’s multiple comparison test using Graphpad Prism v8 and significance indicated: *p<0.05; **p<0.01; ***p<0.001; ****p<0.0001.

Supplementary Figure 6 IL-2 promotes similar increases in HIV replication and TFH viability in TFH infected with CXCR4- or CCR5-tropic HIV. TFH were spinoculated with CXCR4- or CCR5-tropic HIV, labeled with VPD, and cultured at a ratio of 1:1 with uninfected, unlabeled control TFH cells with varying concentrations of IL-2 and 5 μM saquinavir for 5 days. **(A)** Percentages of CXCR4-tropic (left) and CCR5-tropic (right) GFP^+^VPD^+^ TFH were assessed by flow cytometry. **(B)** GFP-MFI was determined in CXCR4-tropic (left) and CCR5-tropic (right) GFP^+^VPD^+^TFH. **(C)** Fold differences of VPD^+^TFH in CXCR4-tropic (left) and CCR5-tropic (right) infected cultures and **(D)** GFP^+^VPD^+^TFH cell counts were determined after five days in culture using absolute count beads and the gating strategy in . Symbols denote individual tonsils (n=3).

Supplementary Figure 7 IL-2 concentration dependent effects of TFR-mediated decrease in HIV replication and TFH viability are similar in TFH infected with CXCR4- or CCR5-tropic HIV. TFH were spinoculated with CXCR4- or CCR5-tropic HIV, labeled with VPD, and cultured at a ratio of 1:1 with uninfected, unlabeled TFH (Control) or TFR for 5 days in the absence or presence of 10, 30, or 100 IU/ml IL-2 and 5 μM saquinavir. Percentages of CXCR4-tropic (left) and CCR5-tropic (right) GFP^+^VPD^+^TFH **(A)**, GFP MFI **(B)**, total VPD^+^TFH cell counts **(C)**, and GFP^+^VPD^+^TFH cell counts **(D)** were quantified by flow cytometry using absolute counting beads and the gating strategy in (n=4).

Supplementary Figure 8 TFR expression of CD39, GITR, and LAG-3 in absence or presence of IL-2. TFH were spinoculated with CXCR4-tropic HIV, labeled with VPD, and cultured at a 1:1 ratio with unlabeled, uninfected TFR for 5 days with 5 μM saquinavir in the absence or presence of 10, 30, or 100 IU/ml IL-2. Percentages of TFR expressing CD39 **(A)**, GITR **(B)**, or LAG-3 **(C)** were quantified *via* flow cytometry (n=6). Statistical analyses were performed using Friedman test and Dunn’s multiple comparison test using Graphpad Prism v8 and significance indicated: *p ≤ 0.05, **p ≤ 0.01.
