# Supplementary figures and images for: Follicular regulatory T cells eliminate HIV-1-infected follicular helper T cells in an IL-2 concentration dependent manner
Source: Front Immunol. 2022 Nov 7;13:878273. doi: 10.3389/fimmu.2022.878273 (PMC9676968; doi:10.3389/fimmu.2022.878273)

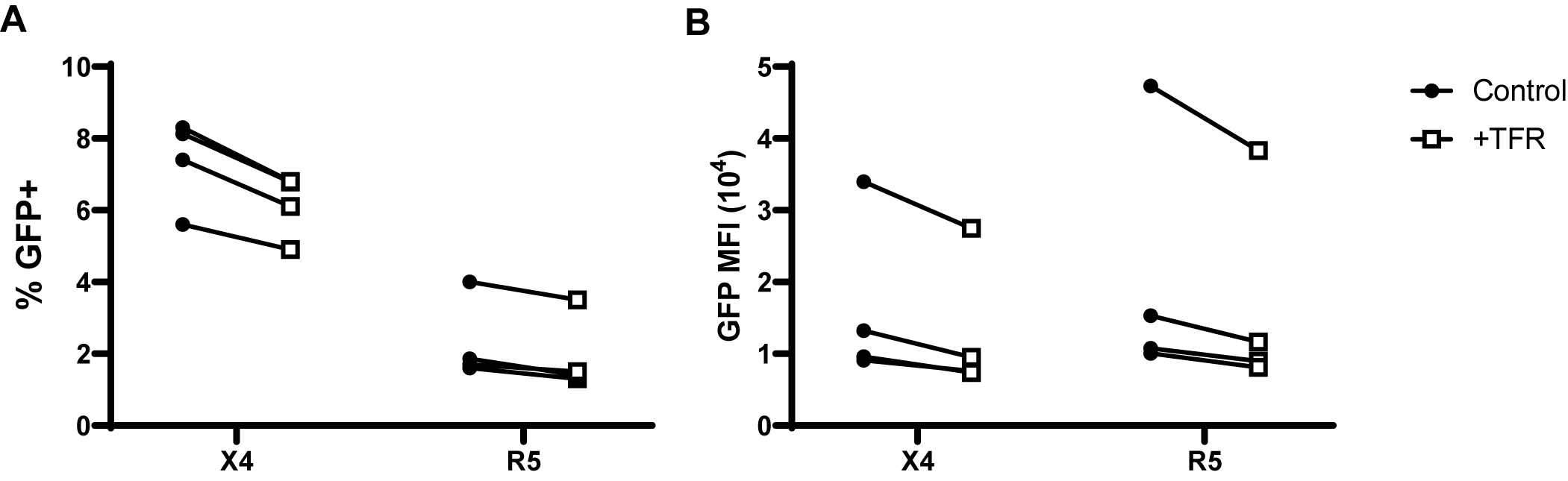

Supplement: Supplementary Figure 1 — TFR cause a similar reduction of HIV replication in TFH infected with CXCR4- or CCR5-tropic HIV. TFH were spinoculated with CXCR4- or CCR5-tropic HIV, labeled with VPD, and cultured with uninfected, unlabeled TFH or TFR in media supplemented with 10 IU/ml IL-2 and 5 μM saquinavir. (A) Percent GFP+ of VPD+ TFH and (B) GFP MFI of GFP+VPD+ TFH were determined after 5 days by flow cytometry using the gating strategy in (n=4). [file Image_1.tif]

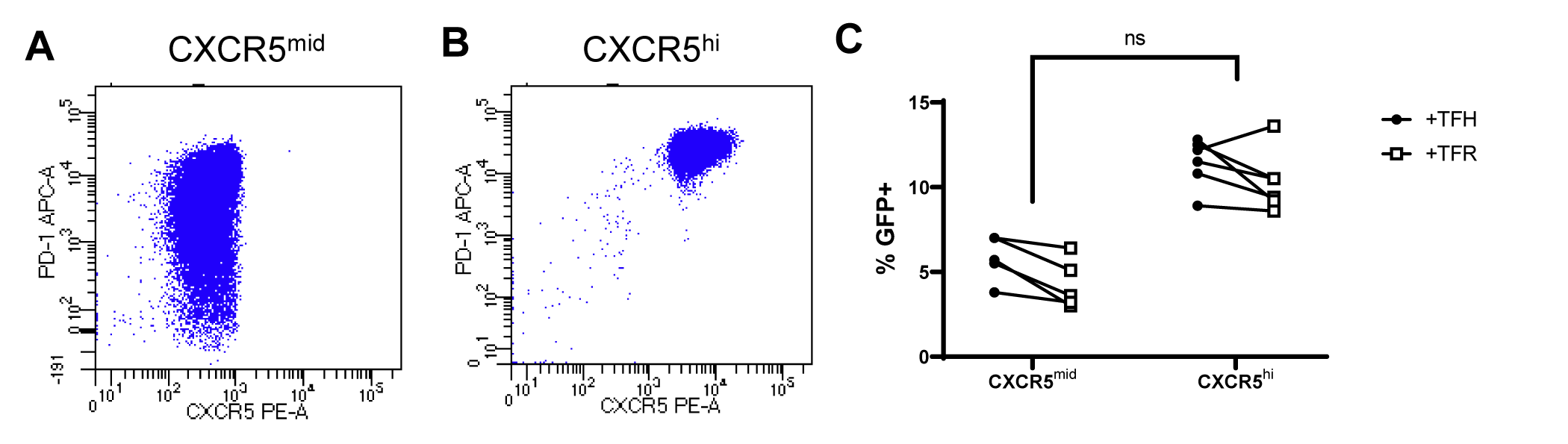

Supplement: Supplementary Figure 2 — TFR reduce CXCR4-tropic HIV replication in CXCR5mid and CXCR5hi TFH. A representative post sort analysis showing CXCR5 and PD-1 expression on (A) CXCR5mid and (B) CXCR5hi TFH after sorting CD4 enriched disaggregated tonsil cells. (C) CXCR5mid and CXCR5hi TFH were spinoculated with CXCR4-tropic HIV, labeled with VPD, and cultured at a 1:1 ratio with unlabeled TFH or TFR in media containing 10 IU/ml IL-2 and 5 μM saquinavir. Percentages of GFP+VPD+ TFH were determined after five days by flow cytometry using the gating strategy in (n=6). Statistical analysis was performed using 2-way ANOVA using Graphpad Prism v8 and significance indicated: ns not significant. [file Image_2.tif]

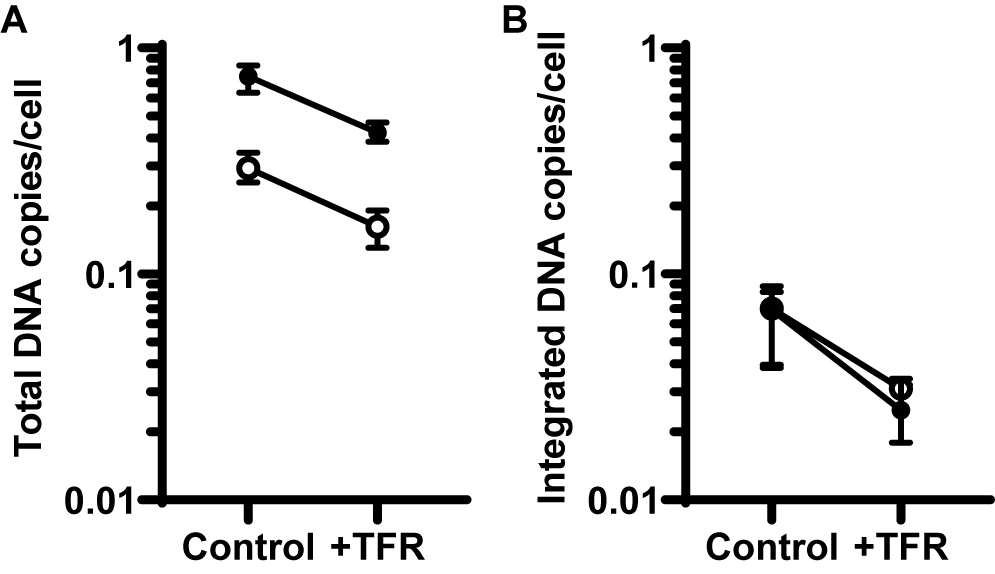

Supplement: Supplementary Figure 3 — TFR reduce total and integrated HIV DNA in TFH. TFH isolated from tonsils were spinoculated with CXCR4-tropic HIV, labeled with VPD, and cultured at a ratio of 1:1 with uninfected, unlabeled TFH (control), or TFR for 5 days in media supplemented with 10 IU/ml IL-2 and 5 μM saquinavir. DNA was isolated from live sorted TFH. Cellular DNA, total HIV DNA (A), and integrated HIV DNA (B) were determined using QPCR (n=2). [file Image_3.tif]

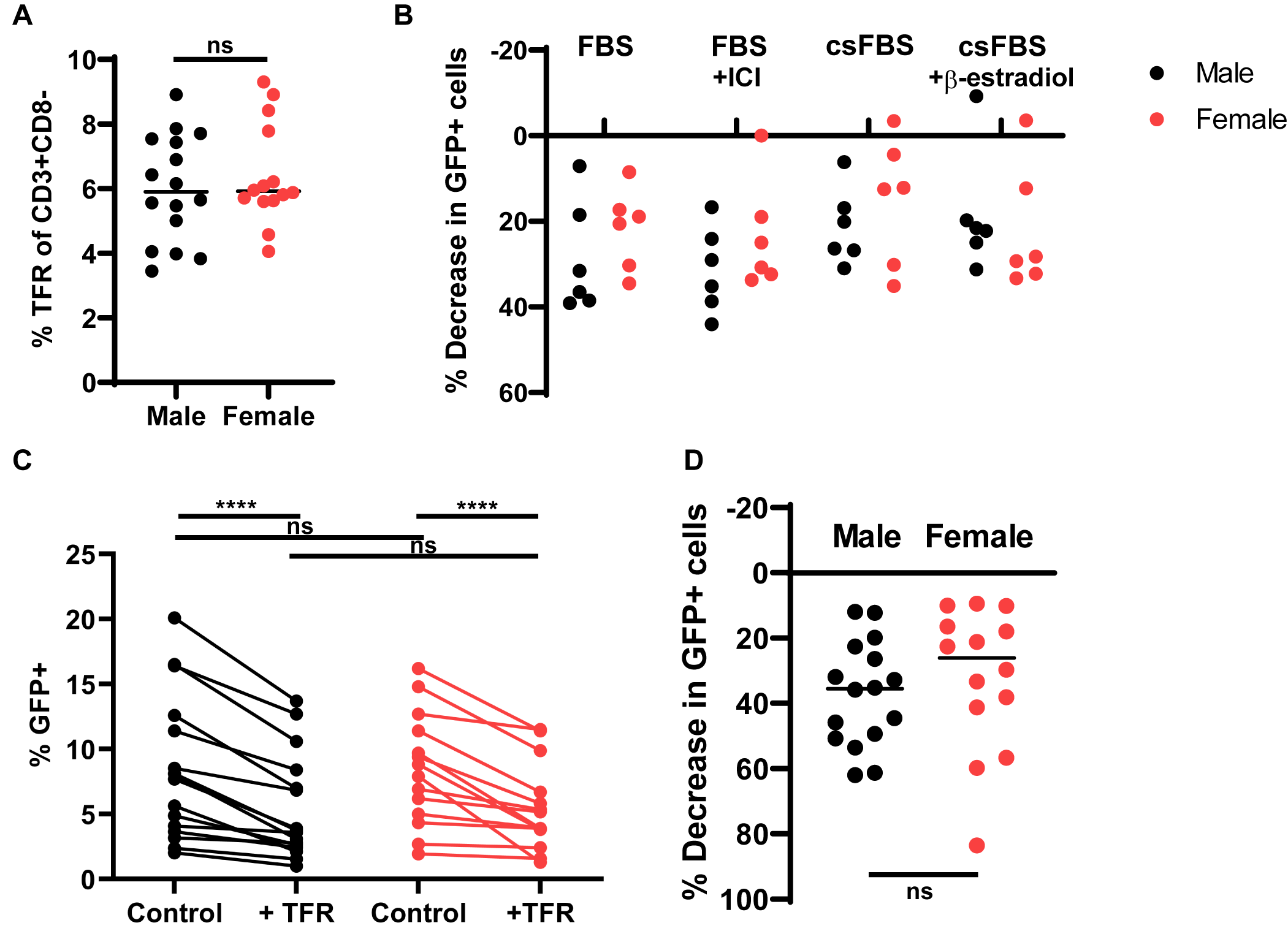

Supplement: Supplementary Figure 4 — Sex differences were not observed in percentages of TFR or TFR mediated inhibition of HIV replication in TFH. (A) Percentages of TFR from tonsil CD3+CD8- cells stained as in were evaluated for sex differences (males, n=16; females, n=14). Statistical analysis was performed using Mann-Whitney test using Graphpad Prism v8 and significance indicated: ns not significant. (B) TFH isolated from tonsils of six males and six females were spinoculated with CXCR4-tropic HIV GFP reporter virus, labeled with VPD and cultured at a ratio of 1:1 with uninfected, unlabeled TFH, or TFR for five days in media supplemented with 10 IU/ml IL-2, saquinavir, and either FBS, FBS supplemented with an estrogen receptor inhibitor (ICI), charcoal stripped FBS (csFBS), or csFBS supplemented with β-estradiol. The decrease in percentages of GFP+ cells was measured for each condition. (C) TFH and TFR were sorted from subjects in (A), spinoculated, labeled, and cultured as indicated in (B) with the exception that all cultures were supplemented with FBS, 10 IU/ml IL-2, and saquinavir. Percent GFP+ of VPD+ TFH was determined after five days using the gating strategy in . Statistical analysis was performed using 2-way ANOVA and Sidak’s multiple comparison test using GraphPad Prism v8 and significance indicated: ns, not significant; ****p<0.0001. (D) Sex differences in percent decrease of GFP+ in cultures shown in (C) were quantified and statistical analysis was performed using Mann-Whitney test using Graphpad Prism v8 and significance indicated: ns not significant. [file Image_4.tif]

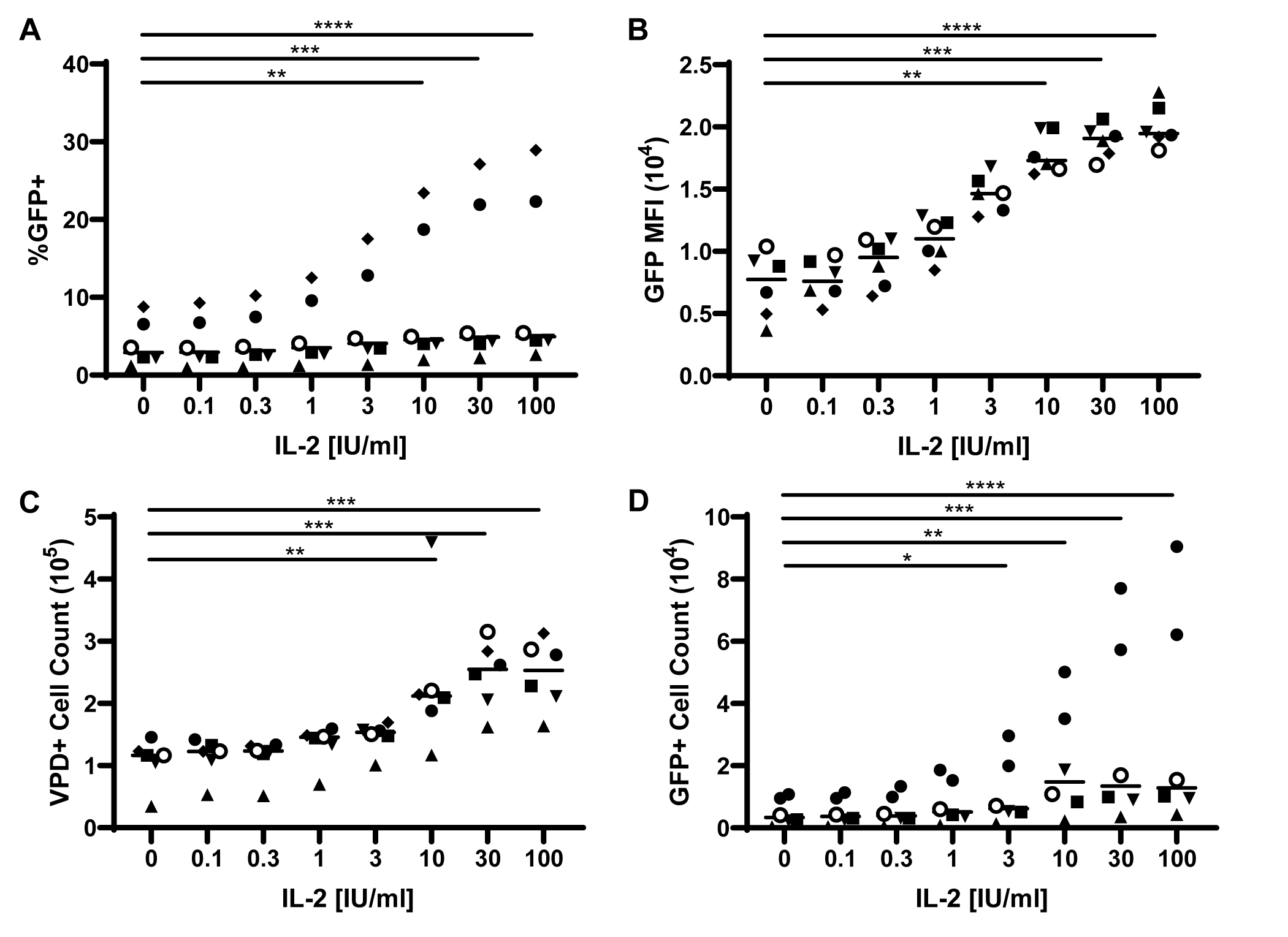

Supplement: Supplementary Figure 5 — Raw data from showing that IL-2 promotes HIV replication and TFH viability. TFH were spinoculated with CXCR4-tropic HIV, labeled with VPD, and cultured at a ratio of 1:1 with uninfected, unlabeled control TFH cells with varying concentrations of IL-2 and 5 μM saquinavir. (A) Percentages of GFP+VPD+ TFH, (B) GFP MFI, (C) VPD+TFH and (D) GFP+VPD+TFH cell counts were determined by flow cytometry after five days in culture using the gating strategy in . Symbols denote individual tonsils (n=6). Statistical analyses were performed with Friedman’s test and Dunn’s multiple comparison test using Graphpad Prism v8 and significance indicated: *p<0.05; **p<0.01; ***p<0.001; ****p<0.0001. [file Image_5.tif]

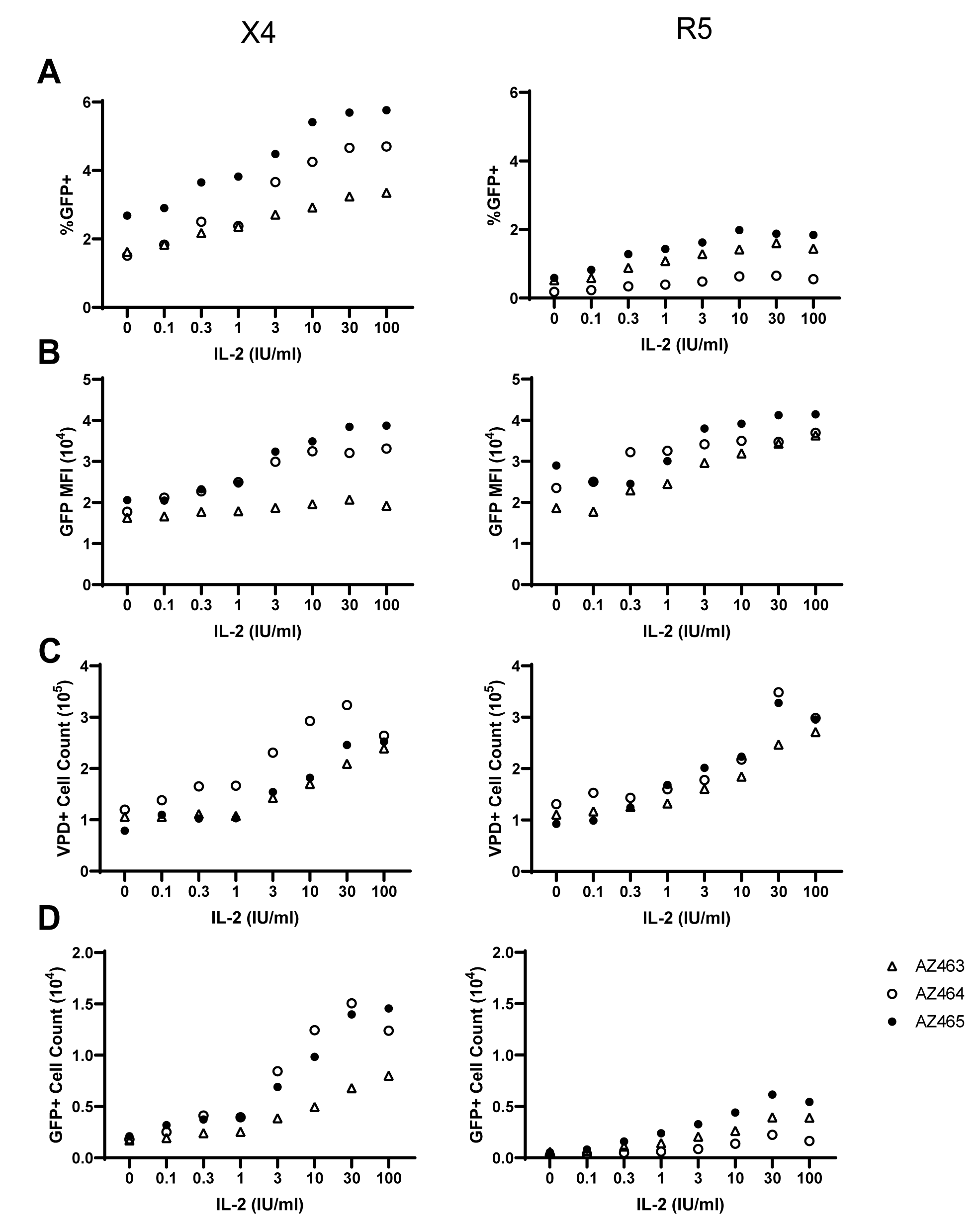

Supplement: Supplementary Figure 6 — IL-2 promotes similar increases in HIV replication and TFH viability in TFH infected with CXCR4- or CCR5-tropic HIV. TFH were spinoculated with CXCR4- or CCR5-tropic HIV, labeled with VPD, and cultured at a ratio of 1:1 with uninfected, unlabeled control TFH cells with varying concentrations of IL-2 and 5 μM saquinavir for 5 days. (A) Percentages of CXCR4-tropic (left) and CCR5-tropic (right) GFP+VPD+ TFH were assessed by flow cytometry. (B) GFP-MFI was determined in CXCR4-tropic (left) and CCR5-tropic (right) GFP+VPD+TFH. (C) Fold differences of VPD+TFH in CXCR4-tropic (left) and CCR5-tropic (right) infected cultures and (D) GFP+VPD+TFH cell counts were determined after five days in culture using absolute count beads and the gating strategy in . Symbols denote individual tonsils (n=3). [file Image_6.tif]

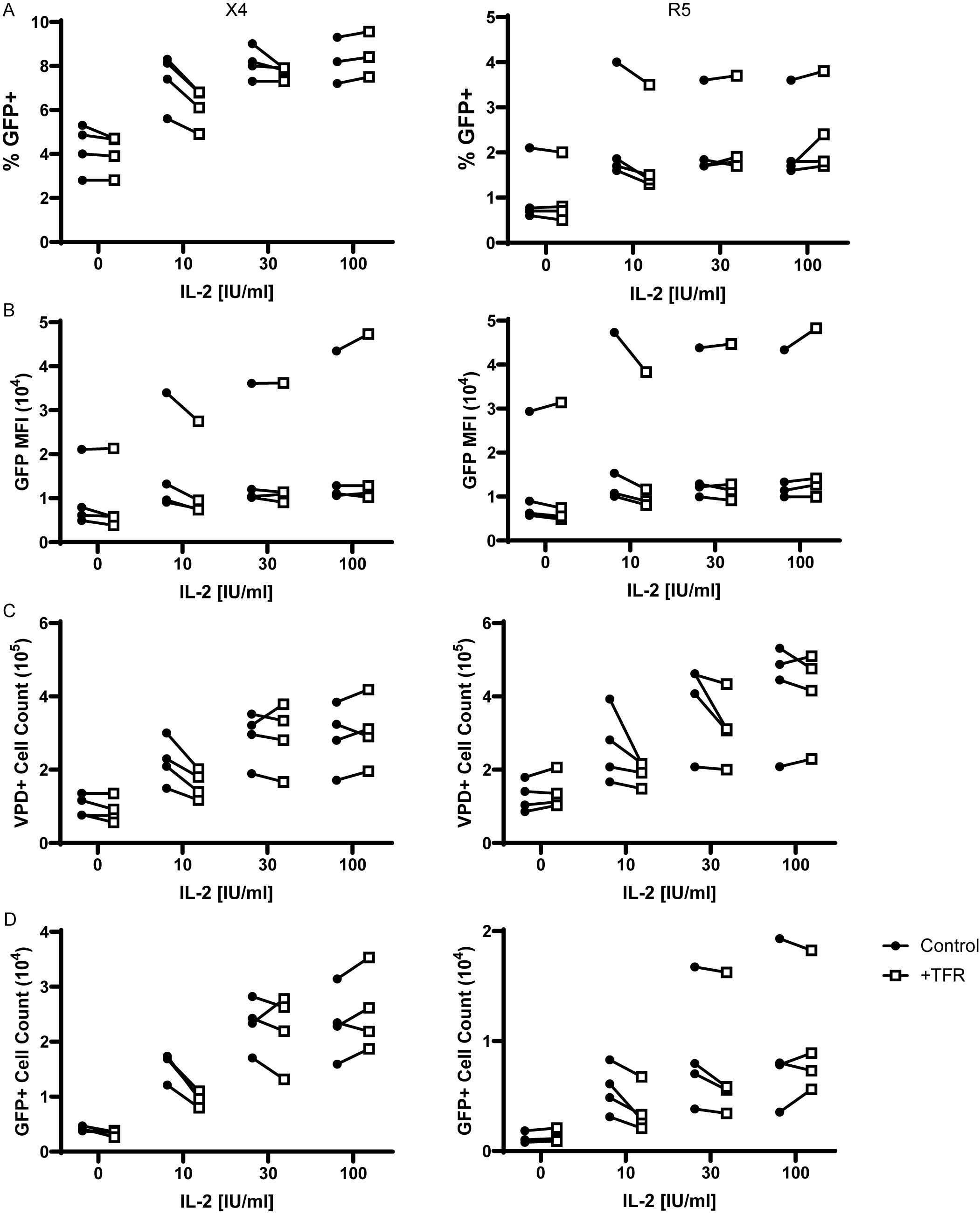

Supplement: Supplementary Figure 7 — IL-2 concentration dependent effects of TFR-mediated decrease in HIV replication and TFH viability are similar in TFH infected with CXCR4- or CCR5-tropic HIV. TFH were spinoculated with CXCR4- or CCR5-tropic HIV, labeled with VPD, and cultured at a ratio of 1:1 with uninfected, unlabeled TFH (Control) or TFR for 5 days in the absence or presence of 10, 30, or 100 IU/ml IL-2 and 5 μM saquinavir. Percentages of CXCR4-tropic (left) and CCR5-tropic (right) GFP+VPD+TFH (A), GFP MFI (B), total VPD+TFH cell counts (C), and GFP+VPD+TFH cell counts (D) were quantified by flow cytometry using absolute counting beads and the gating strategy in (n=4). [file Image_7.tif]

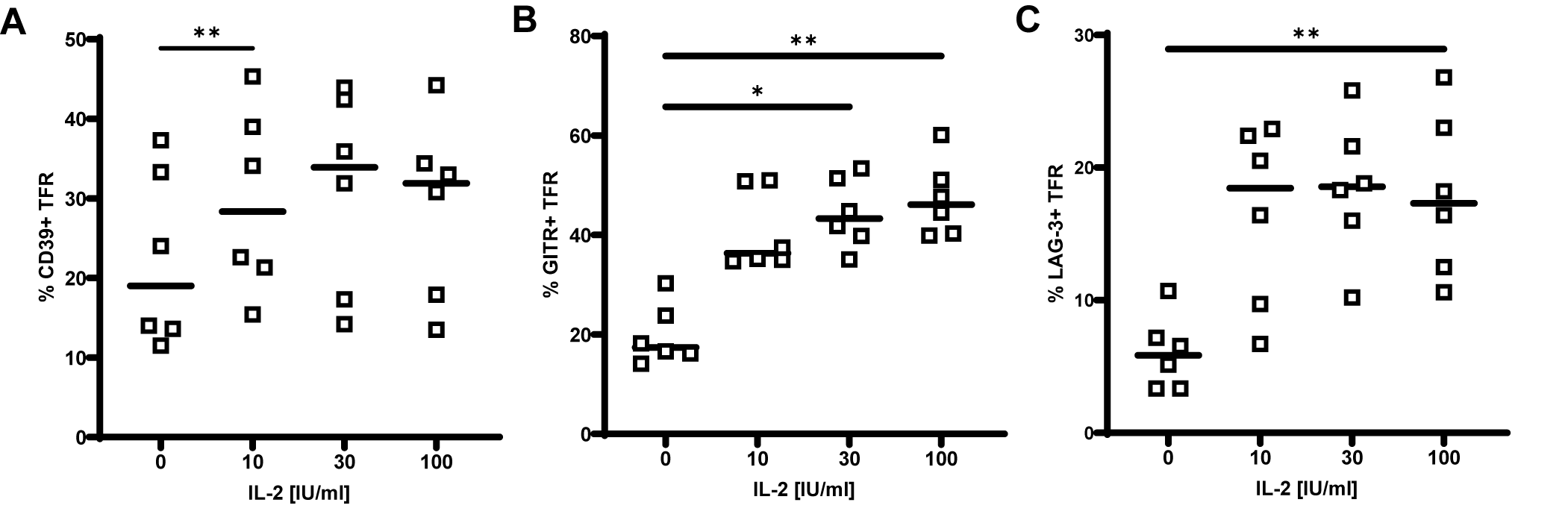

Supplement: Supplementary Figure 8 — TFR expression of CD39, GITR, and LAG-3 in absence or presence of IL-2. TFH were spinoculated with CXCR4-tropic HIV, labeled with VPD, and cultured at a 1:1 ratio with unlabeled, uninfected TFR for 5 days with 5 μM saquinavir in the absence or presence of 10, 30, or 100 IU/ml IL-2. Percentages of TFR expressing CD39 (A), GITR (B), or LAG-3 (C) were quantified via flow cytometry (n=6). Statistical analyses were performed using Friedman test and Dunn’s multiple comparison test using Graphpad Prism v8 and significance indicated: *p ≤ 0.05, **p ≤ 0.01. [file Image_8.tif]
